# Pearls of experience for safe and efficient hospital practices in otorhinolaryngology—head and neck surgery in Hong Kong during the 2019 novel coronavirus disease (COVID-19) pandemic

**DOI:** 10.1186/s40463-020-00427-4

**Published:** 2020-05-15

**Authors:** Ryan H. W. Cho, Zenon W. C. Yeung, Osan Y. M. Ho, Jacky F. W. Lo, Alice K. Y. Siu, Wendy M. Y. Kwan, Zion W. H. To, Anthony W. H. Chan, Becky Y. T. Chan, Kitty S. C. Fung, Victor Abdullah, Michael C. F. Tong, Peter K. M. Ku

**Affiliations:** 1 Department of Otorhinolaryngology—Head and Neck Surgery, United Christian Hospital and Tseung Kwan O Hospital, 2 Po Ning Lane, Tseung Kwan O, New Territories Hong Kong; 2 Department of Speech Therapy, Prince of Wales Hospital, Sha Tin, Hong Kong; 3 Department of Pathology, United Christian Hospital, Kwun Tong, Hong Kong; 4 Department of Otorhinolaryngology—Head and Neck Surgery, The Chinese University of Hong Kong, Sha Tin, Hong Kong

## Abstract

The 2019 novel coronavirus disease (COVID-19) epidemic originated in Wuhan, China and spread rapidly worldwide, leading the World Health Organization to declare an official global COVID-19 pandemic in March 2020. In Hong Kong, clinicians and other healthcare personnel collaborated closely to combat the outbreak of COVID-19 and minimize the cross-transmission of disease among hospital staff members. In the field of otorhinolaryngology—head and neck surgery (OHNS) and its various subspecialties, contingency plans were required for patient bookings in outpatient clinics, surgeries in operating rooms, protocols in wards and other services. Infected patients may shed severe acute respiratory syndrome coronavirus-2 (SARS-CoV-2) particles into their environments via body secretions. Therefore, otolaryngologists and other healthcare personnel in this specialty face a high risk of contracting COVID-19 and must remain vigilant when performing examinations and procedures involving the nose and throat. In this article, we share our experiences of the planning and logistics undertaken to provide safe and efficient OHNS practices over the last 2 months, during the COVID-19 pandemic. We hope that our experiences will serve as pearls for otolaryngologists and other healthcare personnel working in institutes that serve large numbers of patients every day, particularly with regard to the sharing of clinical and administrative tasks during the COVID-19 pandemic.

## Introduction

The 2019 novel coronavirus disease (COVID-19) epidemic originated in Wuhan, a province of China, and spread rapidly throughout the world thereafter. In March 2020, the World Health Organization (WHO) officially declared COVID-19 a pandemic. The 70th situation report issued by the WHO on March 30, 2020 listed 693,224 confirmed COVID-19 cases and 33,106 deaths worldwide, and these statistics encompassed 190 countries, areas and territories in China, Southeast Asia, the Middle East, Europe, the United States and Africa [1]. At the time of preparing this article, although the mortality rate of COVID-19 in China (4.3%) was lower than the rates associated with severe acute respiratory syndrome (SARS) and Middle East respiratory syndrome (MERS) (9.19 and 34.4%, respectively) [2, 3], the global mortality caused by COVID-19 had exceeded mortality during the 2003 SARS outbreak (789 cases) by more than 40 times [4].

Sadly, an otolaryngologist who worked at a hospital in Wuhan, China was infected by SARS-coronavirus-2 (SARS-CoV-2), the causative pathogen of COVID-19, and died in January 2020. By the time of preparation of this article, 2 more otolaryngologists in the U.K. had become infected via transmission from local patients and died of COVID-19, and at least 20 otolaryngologists in Iran had been admitted to the hospital with COVID-19. Many more similar incidents have been reported in European countries [5]. Consequently, many additional healthcare personnel have been quarantined due to close contact with the infected otolaryngologists.

The most commonly reported symptoms of COVID-19 include fever, cough that may be productive, dyspnea and myalgia with fatigue [6]. However, patients suffering from COVID-19 may also present with symptoms of upper respiratory tract infection, such as a sore throat (5–14%) and/or rhinorrhea (4%) [7, 8]. Carriers of COVID-19 may shed a heavy viral load into the environment even if they are asymptomatic or present with very subtle symptoms [9, 10]. Recently, anosmia, hyposmia and dysgeusia, which affect patients’ senses of smell and taste, were identified as symptoms of COVID-19, with high prevalence in Europe, Korea and China [11]. Patients who develop a symptom related solely to their sense of smell or taste before developing respiratory symptoms consistent with COVID-19 may seek consultation with otolaryngologists and receive treatment for rhinitis or neuritis. An asymptomatic patient may still have infectious potential, therefore it is important to have a high level of suspicion and adopt safe practices and precautions.

In 2003 outbreak of SARS in Hong Kong, the initial phase of outbreak began in Prince of Wales Hospital with a carrier of coronavirus in a medical ward causing widespread infection to patients and medical staff through the use of nebulizer for bronchodilators which facilitated the transmission of the virus through aerosol spread. This alerted the precaution of drug administration and aerosol generating procedures in subsequent outbreak of viral disease and the application of appropriate personal protective equipment for safety of healthcare personnel. At the beginning of the outbreak of COVID-19, all suspected and confirmed cases were admitted to hospital for COVID-19 test and those patients with negative result were discharged. This created a considerable workload to Hong Kong public healthcare system and suspension of non-urgent service. With a large number of the suspected cases returning to Hong Kong from Mainland China, Europe, United States and Asian countries with high prevalence of COVID-19, all people were quarantine at home or isolation camps for observation. Only those with subsequent symptoms after arrival to Hong Kong or suspected history and positive test for COVID-19 were admitted to hospital. This had significantly reduced the congestion in the public healthcare system and preserved the capacity to see those patients in need of treatment.

In this article, we share our experience of planning and logistics over a 2-month period to achieve safe and efficient OHNS hospital practices through during the COVID-19 pandemic. In particular, we discuss the clinical and administrative issues encountered during the outbreak. We hope that our experiences will provide pearls for otolaryngologists and physicians in other clinical specialties who work together at institutions that serve large numbers of patients every day.

## Health education and sharing regarding infection control during the COVID-19 outbreak

The provision of health education for healthcare personnel played a very important role at the beginning of the COVID-19 outbreak, when information about the disease was scarce and official guidelines for practice, including otolaryngology practice, were not yet available. Health seminars on COVID-19, which were organized by the infection control team to all hospital staff on daily basis across the whole month, provided a direct platform from which to educate healthcare personnel about the virus, its mode of transmission, the course of the disease, management and the mortality rate. These seminars also enabled otolaryngologists and their nursing staff to share information with the infection control team, particularly regarding proper concepts of infection control in terms of hand hygiene, the donning and doffing of personal protective equipment, the handling of suspected or confirmed cases of COVID-19, contact tracing, the proper disposal of clinical waste and the handling of specimens for any laboratory investigations. This was particularly important, as the OHNS specialty involves a large number of surgical procedures targeting the nose, pharynx and airway, which are performed in clinics, wards and operating rooms. These procedures are often aerosol and respiratory droplet generating.

## Measures to minimize COVID-19 transmission at OHNS services

### Specialty outpatient clinic (SOPC)

Since the first week of February 2020, special arrangement was made to vigorously reduce patient flow to minimize the risk of cross-transmission and preserve manpower for deployment to other clinical teams. Specifically, the outpatients’ visits were reduced to 10–20% of the normal services to ensure that only those with urgent and semi-urgent conditions, such as head and neck cancers, airway emergencies, head and neck abscesses, acute otitis media, acute mastoiditis, complicated sinusitis, sudden hearing loss, facial paralysis or facial trauma, would be seen. During a 6-week period, the OHNS clinic rescheduled more than 2800 cases with no complaints; only a minority of patients wanted to keep their original appointments, whereas more than 95% of patient visits were successfully rescheduled. Drug refills were provided to stable patients without consultation by an otolaryngologist, depending on each patient’s clinical condition, and new follow-up appointments were offered. Patients were triaged at clinic fulfilling the FTOCC criteria: **F**ever, **T**ravel history, **O**ccupational exposure, **C**ontact history and **C**lustering phenomenon were segregated and managed in a separate room with surgical mask on while calling the infection control team for assistance. The travel history to high risk areas was updated in response to the evolving pandemic according to our local guideline from the Centre for Health Protection.

### OHNS endoscopic examination

In OHNS, endoscopy is a high-risk procedure that can induce intra-procedural coughing, sneezing and choking. In our institutes, our group of otolaryngologists also reached a consensus for definitions of several common procedures associated with a potential risk of aerosol generation (Table 1). As a negative-pressure airborne infection isolation room (AIIR) was not available at our clinic, endoscopic procedures were performed in a designated room equipped with an IQAir® HealthPro® 100 (Incen AG, Switzerland) . This device contains a HEPA class H13 filtration system that can filter 99.97% of all particles > 0.3 μm. Additionally, patients were asked to wear a surgical mask at all times before entry to the hospital area, except during the examination to minimize the dispersal of droplet nuclei during patient transfer [12]. In the clinic, all of the fiberoptic endoscopes were labeled with code numbers that were recorded for contact tracing. We strongly preferred to perform endoscopic procedures while wearing high-level personal protective equipment (PPE) (e.g., face shield, N95 respirator, long-sleeved disposable fluid-resistant gown and disposable gloves) (Table 2). From July to December 2019, our clinic performed an average 1890 of fiberoptic endoscopy procedures and 292 rigid endoscopy procedures per month. From February to March 2020, however, the rescheduling of services and very conservative provision of endoscopy examinations reduced the number of fiberoptic and rigid endoscopy procedures to only 134 and 47 per month, respectively, which corresponded to service reductions of 93 and 84%, respectively.

**Table 1:** Procedures and examinations that potentially generate aerosols during the practice of otorhinolaryngology - head and neck surgery

Procedures	Related Risk
Flexible and rigid laryngoscopy	Potential aerosol generation
Nasoendoscopy	Droplet generation by sneezing
Open suction of oral, nasal and aural cavities	Potential aerosol generation
Change of tracheostomy	Potential aerosol generation
Endoscopic guided insertion of feeding tube	Potential aerosol generation by coughing and gag reflex
Tracheoesophageal prosthesis	Potential aerosol generation
Fiberoptic endoscopic evaluation of swallowing	Potential aerosol generation by coughing during aspiration

**Table 2:** Recommended personal protective equipment in different areas of the hospitals and during medical procedures

	Aerosol-generating Procedures	Consultation Room	Clinical Area
Hand Hygiene	Yes	Yes	Yes
Mask	N95	N95	Surgical Mask
Gown	AAMI Level 3	AAMI Level 3	AAMI Level 1
Disposable Gloves	Yes	Yes	Yes
Eye Protection	Goggles/Face Shield	Goggles/Face Shield	Eye Visor
Hair Cover	Optional	Optional	Optional

*AAMI* Association for the Advancement of Medical Instrumentation

### Application of telemedicine

The hospital adopted the strategy of video consultation via the Zoom video communications software platform. This technology allowed clinicians to see some patients who did not require an extensive physical examination. Video consultation was especially suitable for the follow-up of patients with nasal symptoms, hearing impairments, tinnitus, treated sleep apnea, hoarseness with benign causes, dizziness and some facial plastic conditions. Our preliminary trial of this technology was promising, with 28 preoperative and postoperative patients reporting excellent overall satisfaction with a mean score of 9.1/10, effective communication 9.2/10, satisfactory consultation experience 9.2/10 and expectation fulfilled 9.0/10.

### In-patient arrangements and emergency surgery

**Fig. 1:**
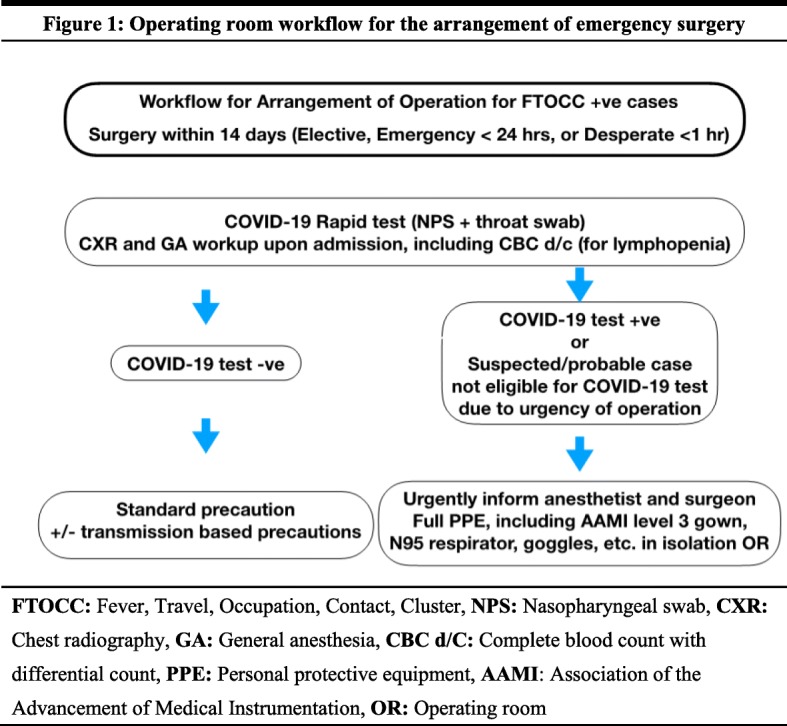
Operating room workflow for the arrangement of emergency surgery

Many ENT examinations and procedures are aerosol-generating (e.g., incision and drainage of peritonsillar abscess, hemostasis of epistaxis). Therefore, healthcare personnel in the ward were required to wear high-level PPE when examining any patients. The number of staff in the ward procedure room was strictly limited to reduce the potential exposure to SARS-CoV-2, and a team of one otolaryngologist with one or two healthcare assistants was preferred. Patients who required urgent consultations with the Accident and Emergency or other clinical departments and presented with fever and respiratory symptoms were subjected to a COVID-19 polymerase chain reaction (PCR) test unless their situation was desperate (Fig. 1). The turnaround time for the COVID-19 PCR test was within 6 h. Confirming a patient’s COVID-19 status was an important step towards minimizing the risk of infection faced by all healthcare personnel in the specialty of OHNS. A good protocol to facilitate the tracing of staff who had had close contact with a confirmed COVID-19 patient was also important (Table 3).

**Table 3:** Contact tracing management in clinics and wards containing confirmed patients with COVID-19^a^

	Close Contacts	Other Contacts
**Healthcare workers (HCWs)**	HCW who has cared for the confirmed case without using appropriate PPE during the procedures	
**In-patients in general wards**^**a**^	1. In-patients who had face-to-face contact for > 15 min with the confirmed cases, regardless of surgical mask use OR 2. In-patients who had shared a cubicle with the confirmed case for > 2 h, regardless of surgical mask use	
**Visitors**	Visitors who had stayed in the same cubicle without a surgical mask for > 15 min	Visitors who had stayed in the same cubicle for < 15 min or had worn a surgical mask
**Quarantine**	Quarantine for 14 days after the last exposure	Not required
**Medical surveillance**	Follow-up medical surveillance for 14 days	28 days after exposure to the confirmed case

Surveillance wards/cubicles are excluded

^a^Reference: Hospital Authority Communication Kit – COVID-19

### Operation room arrangement

To reduce patient flow and reserve hospital beds and staff during the COVID-19 outbreak, the number of elective OHNS operations was reduced to a minimum. A total of 112 elective surgeries were cancelled or postponed in February and March 2020. Only essential elective operations involving head and neck cancer surgery or work-up, airway management and infective conditions were maintained. Using this approach, we reduced our elective surgeries by 80% without complaint.

## Special considerations in OHNS departments during the COVID-19 pandemic

### Low supplies of personal protective equipment

An insufficient supply of surgical masks, protective gowns, face shields and N95 respirators posed a real threat to all healthcare personnel during the period under study, as the demand for these items far exceeded the global supply due to an interruption of the supply chain [13]. Consequently, our department adopted the extended use of PPE such as N95 respirators and protective gowns [14]. Specifically, healthcare personnel used only one N95 respirator and protective gown during the same session of clinical duty, irrespective of the number of patients who were seen, provided that the PPE was not grossly contaminated by blood or body secretions. Face shields were used to protect the contamination of surgical masks or N95 respirators so that extended use was possible. A plastic apron was worn over the protective gown and disposed if it became contaminated, thus eliminating the need to remove the inner protective gown. These PPE-saving measures were required despite the above-described administrative measures intended to reduce patient visits. Using this strategy, the weekly consumption of isolation gowns, N95 respirators and full-face shields decreased from 163 to 59 pieces, 163 to 47 pieces, 163 to 46 pieces, which represented a reduction of 64, 71 and 72% respectively, relative to the initial levels.

### Safety in endoscopic nasal surgery

Endoscopic nasal surgery was noted to increase the risk of exposure for the surgeons and healthcare personnel in the operating room. Indeed, China Newsweek reported that 14 healthcare personnel were infected with SARS-CoV-2 while assisting with a single endoscopic pituitary surgery [15]. Endoscopic nasal surgical procedures that involve the use of powered endoscopic debriders with irrigation and suction or microdrills can generate aerosols loaded with viable virus particles, which spread in the environment. As reports suggest that aerosols may remain in the air for 3 h, healthcare personnel faced a high risk of exposure if they did not wear high-level PPE [16]. Recommendations for any endoscopic nasal surgery may involve the required routine testing of COVID-19 twice with a 24-h interval to confirm infection negativity before proceeding to surgery, as N95 respirators may not be sufficient to protect healthcare personnel in the operating room [17]. With our initial measures in surgery arrangement and infection control, no otorhinolaryngologists or healthcare workers in Hong Kong had a nosocomial COVID-19 infection.

### Airway management plan for COVID-19 patients

According to a report by the Chinese Center for Disease Control and Prevention that included approximately 44,500 confirmed cases of COVID-19, approximately 5% of the patients surveyed developed critical disease involving respiratory failure, shock or multi-organ dysfunction that required ventilation [18]. Tracheostomy may be required for any patient subjected to prolonged intubation due to respiratory failure associated with COVID-19. During the 2003 SARS outbreak, the OHNS team performed tracheostomy in only 3 (0.87%) of the 343 confirmed cases under treatment at the Prince of Wales Hospital [19]. From previous experience in SARS, the decision on tracheostomy was jointly made by intensivist, anesthesiologist and otorhinolaryngologist for patients intubated for more than 4 weeks in general, as ample time was required for evaluation of possible extubation for recovering patients.

During tracheostomy, the number of healthcare personnel in the operating room should be limited. Senior anesthetists and experienced otolaryngologists should perform the surgery in a negative-pressure AIIR, and all operating room staff members should wear high-level PPE and adopt contact- and airborne-protective measures. Anesthetists should ensure that the patient is fully paralyzed to facilitate a smooth intubation. Two disposable high-efficiency bacterial and viral hydrophobic filters should be placed in the expiratory circuit of the ventilator. Airway suction must be performed in a closed system during the procedure. Moreover, the apnea technique should be adopted during fenestration of the tracheal wall and insertion of the tracheostomy tube. A well-fitting cuffed tracheostomy tube should be inserted with the cuff inflated to prevent air leakage and minimize the frequency of post-operative tube changing. Good communication between the anesthetists and nurses is essential throughout the procedure.

### Head and neck oncologic surgery

In our OHNS department, cancer operations were prioritized according to the aggressiveness of the tumor, presence of complications (e.g., airway obstruction, dysphagia and bleeding) and potential impact on reconstructive methods. Previous studies have clearly documented the high doubling rate of head and neck squamous cell carcinoma, and a wait time longer than 4 weeks may lead to worse prognostic outcomes, such as the development of new lymph node metastasis (20% of cases) and progression according to the Tumor, Node, Metastasis (TNM) classification (16%) [20]. Patients with head and neck squamous cell carcinoma were therefore assigned a high priority, followed by those with high-grade salivary gland cancer and undifferentiated nasopharyngeal carcinoma. In contrast, papillary thyroid cancer, which has a much more favorable prognosis and a 10-year survival rate > 90%, was considered a lower priority [21]. Early operations were allocated to the resection of bulky tumors in the larynx or hypopharynx, as this might avoid complete airway obstruction requiring an emergency tracheostomy. Small oral cavity tumors were also prioritized, as a delay in treatment might necessitate a more complex reconstruction (e.g., free flap reconstruction), which would lead to more significant morbidity, a longer operating time and a longer hospital stay. With the available weekly operative list for patients suffering from head and neck cancers, 9 cases were operated in February and March 2020.

### Pediatric OHNS

Emerging evidence from China indicates that children usually develop mild symptoms of COVID-19 [22]. Moreover, COVID-19 patients as young as 3 days have been reported [23]. In a case series of 10 pediatric patients in China, all of the children recovered spontaneously and did not require oxygen therapy. Nonetheless, the severity of symptoms should not determine the level of precaution, as asymptomatic or mildly symptomatic patients may be as infectious as their more severely affected peers [24]. Pediatric airway procedures or even simple examinations are considered highly likely to generate droplets or aerosols via crying and/or coughing vigorously during a fiberoptic endoscopic examination. Therefore, it is critical to maintain a high level of precaution during pediatric examinations. In this context, the healthcare personnel were required to wear high-level PPE in anticipation of aerosol generation during the examinations and procedures.

### Sleep medicine in OHNS

To save time and reduce the risk of cross-infection during the COVID-19 pandemic, teleconsultations with otorhinolaryngologists and maxillofacial surgeons were arranged for stable patients of the combined sleep surgery clinic [25]. In-patient polysomnography (PSG) studies were replaced by home studies, including Watch-PAT and Nox-2 studies, as these methods have exhibited high clinical correlations with the gold-standard PSG method [26, 27]. Drug-induced sleep nasal endoscopy was suspended as it is an aerosol generating procedure, unless a polymerase chain reaction (PCR) test for SARS-CoV-2 yielded negative results prior to examination.

### Measures in audiology and speech therapy

In audiology practice, contact transmission remains the most common route of COVID-19 transmission [28]. Therefore, audiologists must understand the importance of hand hygiene, demonstrate the appropriate use of PPE for various procedures and remain alert to the cleaning and disinfection of reusable items [29]. During this pandemic, concerns were expressed about pediatric patients, as most did not comply with the instruction to wear a facial mask. Audiologists should wear appropriate PPE for self-protection in any situation. Toys and reusable items handled during a play audiology assessment must be cleaned thoroughly after use.

During speech therapy, the assessment and training of patients with swallowing, speech and voice pathologies may generate aerosols. This risk increased further when working with patients with tracheostomy and laryngectomy, as any training procedures might require suctioning that could induce choking, coughing, sneezing and projectile vomiting. Teleconsultations through video call were used to avoid direct contact and enable the proper evaluation of patients without the need to wear facial masks. Swallowing assessments were performed by video-fluoroscopy, which was a good alternative to the fiberoptic endoscopic evaluation of swallowing because it allowed speech therapists and radiologists to maintain a distance from the patients during the assessments.

## Discussion

COVID-19 is a common enemy worldwide, and to date, more than 690,000 people have been infected by SARS-CoV-2. During the 2003 SARS outbreak, almost 20% of all infected cases involved healthcare workers, which further jeopardized the existing public healthcare system [30]. This situation may be repeated during the COVID-19 pandemic if healthcare facilities do not increase their vigilance in terms of the protective measures used by healthcare workers. In China, nearly 3400 healthcare workers had contracted COVID-19 and 13 had died by early March 2020 [31]. In Italy, at least 2629 healthcare workers had contracted COVID-19 by mid-March 2020, and these cases accounted for 8.3% of all cases in that country [32]. The U.S.A. appears to be the next country at risk, with more than 160,000 confirmed COVID-19 cases as of March 30, 2020. Otolaryngologists work very closely with professionals in many clinical specialties. Accordingly, although healthcare personnel in the specialty of OHNS are highly susceptible to the risk of SARS-CoV-2 infection, their colleagues in partnered specialties must also remain vigilant when seeing patients together with otolaryngologists in any clinical environment.

Having experienced the SARS outbreak in 2003, the residents of Hong Kong were therefore very alert of COVID-19 and adopted escalated personal hygiene habits, including facial mask and hand sanitizer use and avoidance of social gatherings, at an early stage. Accordingly, the number of suspected COVID-19 cases in Hong Kong as of March 29, 2020 was approximately 5798 with only 641 confirmed cases [33]. As we started our contingency plan very early with zealous education of the health care personnel and public, we could attain zero infection rate in our health care workers in the hospitals and no otolaryngologists in Hong Kong were infected. However, the situation could be worsened if outbreaks were not under control in Europe, the U.S.A., Canada, Australia, Southeast Asia and the Middle East. Although the duration of the COVID-19 pandemic cannot be predicted accurately, some experts in Hong Kong claimed that it will not end until a SARS-CoV-2 vaccine becomes commercially available. Therefore, we may need to revise our clinical practice guidelines and protocols for OHNS from the perspective of infection control, as our clinical services cannot be reduced indefinitely in the face of a long pandemic. Tele-consultation provided a good tool to see our patients who could not attend the clinic because of quarantine regulation of Hong Kong, cities lockdown in China or suspension of clinic service in our hospitals. The feedback from patients was excellent as they were very grateful to be seen by their doctors distantly from their home or office through their mobile phones while the hospitals were under intense pressure on manpower and beds in this pandemic. In future practice of ENT, each consultation room would install HEPA filter and electronic endoscopes for nose, throat, and ear examination in order to keep distance from the patients. Negative pressure ventilation should be installed in at least one consultation room to cater for highly suspected or confirmed case of highly infectious viral diseases. Finally, we hope our clinical experiences will be useful to professionals in the specialty of OHNS and related multidisciplinary services during this COVID-19 pandemic.

## Data Availability

Data sharing is not applicable to this article as no datasets were generated or analyzed during the current study.

## Abbreviations

AAMI: Association of the Advancement of Medical Instrumentation
AIIR: Airborne infection isolation room
CBC d/c: Complete blood count with differential count
COVID-19: 2019 novel coronavirus disease
CXR: Chest X-ray
FTOCC: Fever, travel history, occupational exposure, contact history, clustering phenomenon
GA: General anesthesia
HCWs: Healthcare workers
MERS: Middle East respiratory syndrome
NPS: Nasopharyngeal swab
OHNS: Otorhinolaryngology – head and neck surgery
OR: Operating room
PCR: Polymerase chain reaction
PPE: Personal protective equipment
PSG: Polysomnography
SARS: Severe acute respiratory syndrome
SARS-CoV-2: Severe acute respiratory syndrome coronavirus-2
SOPC: Specialty outpatient clinic
TNM: Tumor, node, metastasis
WHO: World Health Organization
